# PW03-032 – Periodic fevers in children

**DOI:** 10.1186/1546-0096-11-S1-A258

**Published:** 2013-11-08

**Authors:** C Harper, C Corlett, G Macdonald, G Worthington, D Porter, D Kelly, S Segal, AJ Pollard, S Atkinson

**Affiliations:** 1 Paediatric Department, University of Oxford, UK; 2 Paediatric Department, Oxford Children's Hospital, UK; 3 NIHR , Oxford Biomedical Research Unit, Oxford, UK

## Introduction

This is a retrospective case review of patients presenting with periodic fevers to the paediatric infectious disease clinic at the Children’s Hospital, Oxford, over a ten year period.

## Objectives

The aim of the study was to characterise a cohort of children presenting with periodic fevers and to determine whether those with PFAPA (periodic fever, aphthous stomatitis, pharyngitis, cervical adenitis syndrome) have unique features compared to other periodic fever syndromes.

## Methods

Hospital records and biochemical data were obtained for patients presenting to the paediatric infectious disease clinic with periodic fever syndromes between 2001 and 2011. 68 patients were identified but 30 were excluded as they had a clear focus of infection or no history of recurrent fever on review of the medical records. The clinical diagnosis, patient demographics, aetiology, fever behaviour, presenting symptoms, biochemical features and response to treatment are described in the final cohort of 38 patients.

## Results

PFAPA was the most prevalent periodic fever syndrome recognised (9 cases, 24%). Other diagnoses included Hyper IgD Syndrome, Familial Mediterranean Fever and Cryopyrin-Associated Periodic Syndrome; 17 (31%) patients remained undiagnosed. All patients were <5yrs at diagnosis and 23 (61%) patients were male. Common presenting symptoms included pharyngitis, cervical adenopathy and abdominal pain. Fever episodes lasted between 3 to 6 days and inflammatory markers were raised during fever episodes. Five (55%) PFAPA cases had a positive family history and six (71%) were treated successfully with tonsillectomy.

## Conclusion

We characterised the clinical and biochemical features of patients presenting with periodic fever syndromes and found a high prevalence of PFAPA compared to other periodic syndromes though in many patients no firm diagnosis was made. The family history in a high proportion of cases strongly implies that genetic determinants of these syndromes should be identified.

## Disclosure of interest

None declared

